# Effects of reminiscence therapy combined with memory specificity training (RT-MeST) on depressive symptoms in older adults: a randomized controlled trial protocol

**DOI:** 10.1186/s12877-023-03967-2

**Published:** 2023-06-29

**Authors:** Yuejin Wu, Xin Zhang, Tianzhuo Yu, Xin Sui, Yuewei Li, Haiyan Xu, Ting Zeng, Xin Leng, Lijing Zhao, Feng Li

**Affiliations:** School of Nursing, Jilin University, 965 Xinjiang Street, Changchun, 130012 China

**Keywords:** Autobiographical memory, Depression, Older people, Reminiscence, Randomized controlled trial

## Abstract

**Background:**

Geriatric depression has become a serious public health problem, and reduced autobiographical memory and increased overgeneral memory, as the main cognitive markers of depression, are not only associated with current depressive symptoms but also associated with the onset and course of depression, which can lead to a range of harms. Economic and effective psychological interventions are urgently needed. The aim of this study is to confirm the effectiveness of reminiscence therapy combined with memory specificity training in improving autobiographical memory and depressive symptoms in older adults.

**Methods:**

In this multicentre, single-blind, three-arm parallel randomized controlled study, we aim to enrol 78 older adults aged 65 years or older with a score of ≥ 11 on the Geriatric Depression Scale, and participants will be randomly assigned to either a reminiscence therapy group, a reminiscence therapy with memory specificity training group or a usual care group. Assessments will be conducted at baseline (T0) as well as immediately post-intervention (T1) and 1 (T2), 3 (T3) and 6 (T4) months post-intervention. The primary outcome measure is self-reported depressive symptoms, measured using the GDS. Secondary outcome measures include measures of autobiographical memory, rumination, and social engagement.

**Discussion:**

We believe that the intervention will play a positive role in improving autobiographical memory and depressive symptoms in older adults. Poor autobiographical memory is a predictor of depression and a major cognitive marker, and improving autobiographical memory is of great significance in alleviating depressive symptoms in older people. If our program is effective, it will provide a convenient and feasible strategy for further promoting healthy ageing.

**Trial registration:**

ChiCTR2200065446.

## Background

Ageing is a global issue. On the one hand, the number of older people is increasing every year, and in 2019, there will be approximately 700 million older people worldwide; the number of people over 80 years old is expected to triple by 2050 [1]. On the other hand, as the human disease spectrum shifts from acute high-mortality infectious diseases to chronic low-mortality diseases, life expectancy has increased dramatically worldwide; however, healthy life expectancy varies across countries and regions [2, 3]. In China, from 2010 to 2020, the proportion of people over 65 years of age rose from 8.2% (111 million) to 18.7% (191 million) and is expected to reach 26.9% (400 million) by 2050 [4], with an average life expectancy of 76.4 years and a healthy life expectancy of approximately 68.7 years, which means that older people will have approximately 8 years of disease-carrying survival [5]. China will face an immense challenge of population ageing in the coming decades, and the physical and psychological health of older people as a public issue needs more attention.

With increasing age, older people have poor physical health, such as a decline in daily activities, poor physical mobility, and the presence of multiple disease comorbidities; psychological problems are also prominent [6, 7]. Due to the sense of uselessness brought about by the decline in physical functions, the change in social roles, social isolation since COVID-19 and worries about the spread of the virus [8], older adults may develop a series of psychological problems such as depression and loneliness, among which the incidence of depression ranks first among psychological disorders in older adults [9, 10], impairing their health and quality of life and resulting in a mortality rate that is 1.8 times higher than that of nondepressed patients. In addition, depressed older adults use more outpatient resources than nondepressed older adults, including frequent appointments and laboratory and radiological tests [11], which brings great economic costs to society [12]. Depressive symptoms in older adults are significantly associated with loneliness [13] and are an independent risk factor for decline in self-rated health [14]. Therefore, cost-effective psychological interventions are urgently needed to improve depressive symptoms in older adults and to effectively prevent further development of depressive symptoms.

There are a number of psychological treatments available to alleviate the symptoms of depression in older adults, such as cognitive behaviour therapy, music therapy, problem-solving therapy, and web-based self-management interventions. However, depression as a mental illness has been extensively studied by psychiatrists. A review of previous psychiatric studies related to geriatric depression found that older adults often have difficulty recalling autobiographical memories (ATMs) of discrete events that occurred at a specific place and time in their lives and instead retrieve overgeneral memories (OGMs)[15]. Autobiographical memory is the storage of personal life experiences, which is crucial to shaping the self and maintaining mental health [16, 17], and studies have shown [18]that when depressed patients are given a cue word, they retrieve fewer specific memories and more overgeneral memories than nondepressed individuals in the control group. OGM is a phenomenon in which it is difficult for individuals to extract specific autobiographical memories; in contrast, it will produce categorical or extended memories [19], which is a cognitive marker of depression. Such an overgeneralized memory style is a vulnerable factor for depression [20]; it is related not only to the current symptoms but also to the onset and course of depression [21, 22], which can lead to a series of harms such as impaired social problem solving, rumination and worsening symptoms of anxiety and depression [23]. In recent studies, psychiatrists have suggested that the treatment effect can be improved by improving this psychopathology-based cognitive marker of depression.

Reminiscence therapy, as a potential psychological intervention to improve depressive symptoms in older adults, has an important therapeutic aspect of enhancing autobiographical memory and is often used interchangeably in the literature due to its similarity to the term “life review intervention” [24]. It enhances older people’s positive affect [25], cognitive functioning [26], social participation [27], quality of life, and ability to adapt to ageing [28] through the recall, narration, and reflection of past experiences. In addition to previous meta-analyses showing the significant effect of reminiscence-based interventions in improving psychological problems and enhancing well-being in older adults [29–31], there are also empirical studies showing that when researchers guided older adults through interventions for reminiscence at different stages of life, the older adults became more resistant to social stress, increased their ability to think and act for themselves, and finally, experienced completeness in the final stages of life [32–34]. However, in terms of the improvement of autobiographical memory, studies have shown that reminiscence therapy is not effective in improving it [35–37], possibly because reminiscence therapy is an inherent normative process of thinking about the past [38], rather than the practice and improvement of specific event details, which cannot significantly improve the effect of autobiographical memory.

Memory-specific training (MeST), as an intervention for improving autobiographical memory in some patients with mood disorders, has a good effect on patients’ difficulties in recalling personal events through the use of clues to recall words. Although MeST is simple and can be implemented singly, the effect is not good [39, 40]; thus, it can also be implemented together with traditional interventions such as reminiscence therapy as an adjunct therapy [40]. Consequently, there have been many studies combining these two therapies.

Currently, reminiscence therapy combined with memory-specific training is mainly used in the psychological treatment of cancer patients in palliative care, and its effectiveness needs further validation, as randomized controlled trials of Life Review Therapy combined with Memory Specificity Training (LRT- MST) by Gitta Kleijn showed no significant differences between the two groups in terms of anxiety, depression, quality of life and autobiographical memory in cancer patients [41, 42]. However, other studies have shown that reminiscence therapy based on autobiographical memory training can be used in future studies to investigate available, cost-effective, low-intensity treatment options for patients experiencing mood disorders [43, 44]; thus, further investigation of this therapy is warranted.

Based on the national situation that depressed older people are increasing year by year in China, this study aims to implement a randomized controlled trial of reminiscence therapy combined with memory specificity training in a Chinese context to investigate whether this therapy can reduce rumination and improve depressive symptoms in older people and improve their psychological health by improving autobiographical memory. In this study, it is hypothesized that older people who participate in reminiscence therapy combined with memory specificity training will report improved autobiographical memory specificity, less rumination, and significantly lower levels of depressive symptoms at postintervention and follow-up time points than older adults receiving usual care and reminiscence therapy alone; we further hypothesize that these improvements will be maintained for 6 months after the intervention.

## Methods

This study was designed, performed and reported according to the Standard Protocol Items: Recommendations for Interventional Trials (SPIRIT) guidelines [45].

### Study Design

A multicentre, single-blind, three-arm, randomized controlled trial will be conducted to assess the effect of reminiscence therapy combined with memory specificity training on improving autobiographical memory in older adults and further assess the effect on improving depressive symptoms in older adults. Based on the screening criteria, 78 participants will be recruited from three nursing homes in the provincial capital of China, and participants will be randomly divided into three groups in a 1:1:1 ratio. Participants in the first group will receive a 4-week intervention with reminiscence therapy, participants in the second group will receive a 4-week intervention with reminiscence therapy combined with memory specificity training, and participants in the third group will receive 4 weeks of usual care, followed by a 24-week follow-up.

**Fig. 1:**
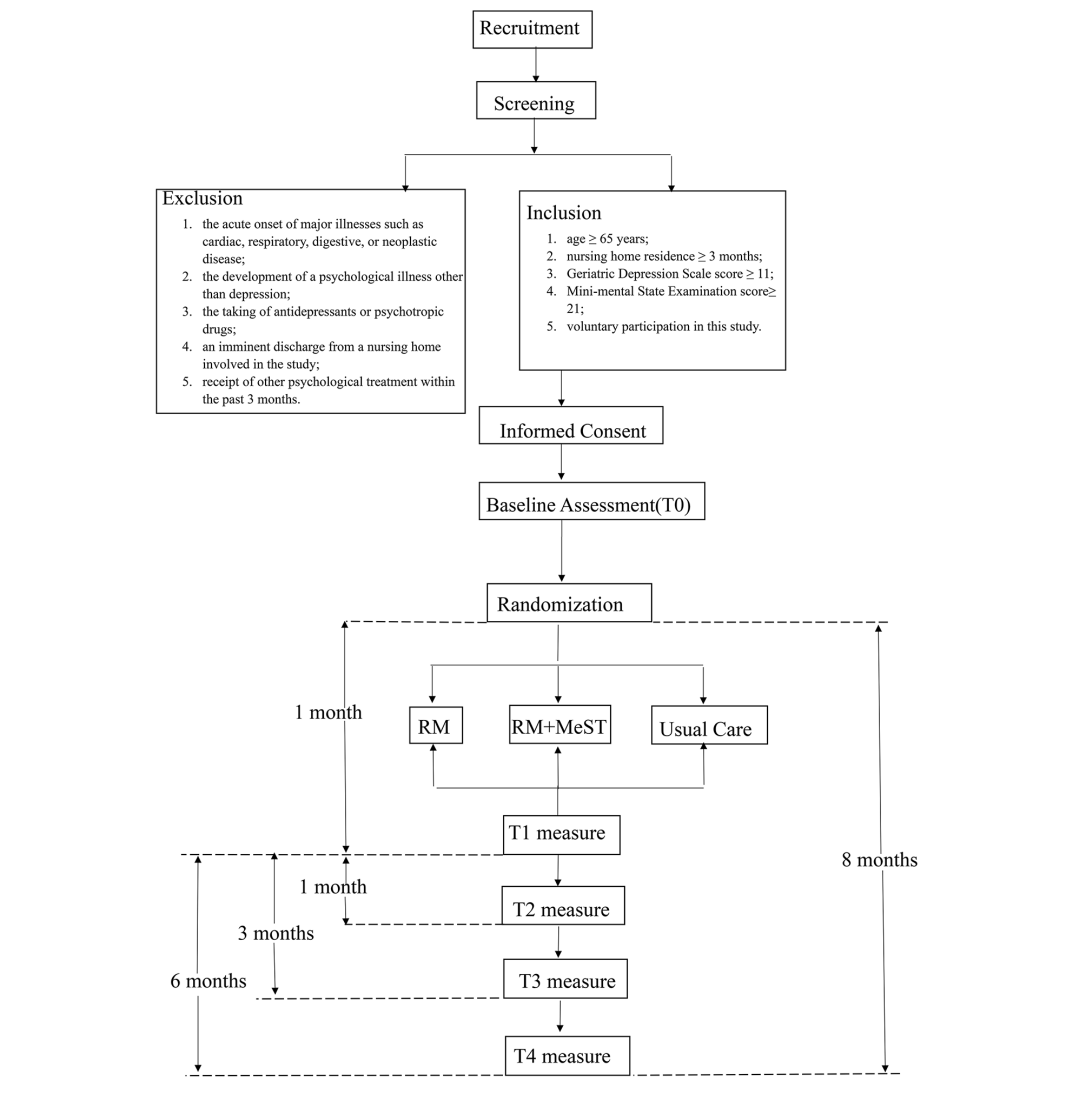
Trial flowchart

Data will be collected at baseline, immediately postintervention, 1 month, 3 months, and 6 months postintervention. The study design is shown in Fig. 1. After the intervention, the same instructions will be given to a third group to ensure that more older adults benefit.

### Participants

#### Eligibility and recruitment

Participants will be recruited from all three nursing homes simultaneously.

Once a nursing home has a sample of 26 subjects, they will proceed to the next step. Inclusion criteria are age ≥ 65 years; nursing home residence ≥ 3 months; Geriatric Depression Scale score ≥ 11; Mini-mental State Examination score ≥ 21; and voluntary participation in this study. Exclusion criteria include the acute onset of major illnesses such as cardiac, respiratory, digestive, or neoplastic disease; the development of a psychological illness other than depression; the taking of antidepressants or psychotropic drugs; an imminent discharge from a nursing home involved in the study; and receipt of other psychological treatment within the past 3 months.

### Sample size calculation

Based on a priori power analysis (G-power3.1.9.7) using a power of 0.90 and error probability of 0.05, with a reference to a literature effect size of 0.8 [46, 47], a sample size of 24 participants per group will be needed. Assuming a 10% drop out rate, a sample size of 78 participants will be initially targeted.

### Randomization and blinding

Among all nursing homes with similar conditions in the capital city of a province in China, three nursing homes will be selected to be included in this study by using the random number table [48], and then the envelope method will be used to determine which nursing home would implement which intervention. After obtaining the nursing homes’ consent to participate, the allocation will be kept concealed by random grouping. This work will be done independently by individuals who are not involved in the study and who keep a strict allocation sheet.

### Intervention

To better tailor the reminiscence content to the actual situation of older people and evoke their emotional resonance, the reminiscence content of this study will be obtained through preinterviews with the participants. The interview outline has been formulated by researchers and revised by experts, and the interview results will be taken as the specific content of the reminiscence of older people in this study. The outline of the interview is shown in Table 1.

**Table 1:** Interview Outline

Reminiscence Stage	Reminiscence content	Points of interest
Childhood (0–18 years)	1、What experiences from your childhood impressed you more? 2、If you recall, what would you most like to recall? 3、What did you do most of the time during your childhood?	Family Study experience Holidays
Young adulthood (18–25 years)	1、What experiences were special to you during your youth? 2、Do you remember what your first job was? 3、If you recall, what would you like to recall most?	Friendship Education Technology change
Adulthood (25–65 years)	1、What experiences during your adulthood were special to you? 2、What were the major turning points? 3、If you recall, what would you like to recall most?	Spouse Parenting Professional experience
Old age (over 65 years)	1、What experiences are special to you at the moment? 2、Do you have any personal feelings about life? 3、Describe a decisive event in your life	Philosophy of life Looking back

#### Reminiscence therapy

The group will implement group reminiscence therapy with 4 reminiscence phases. Each subgroup will consist of 1 implementer and 6–7 participants and will focus on 1 growth phase once a week for 90 min each time. The implementer will be responsible for guiding and encouraging the group members to freely recall past experiences and ensuring that each participant speaks for at least 10 min. The intervention details are shown in Table 2.

**Table 2:** Intervention details

Sessions	Content
1st (week 1)	**Life stage**: Childhood (0–18 years) **Topic**: family, study experience, holidays etc. **Members**: 1 implementer and 6–7 participants. **Duration**:90 min. **Method**: The implementer will be responsible for guiding and encouraging the group members to freely recall past experiences and ensuring that each participant speaks for at least 10 min.
2nd (week 2)	**Life stage**: Young adulthood (18–25 years) **Topic**: friendship, education, technology change etc. **Members**: 1 implementer and 6–7 participants. **Duration**:90 min. **Method**: The implementer will be responsible for guiding and encouraging the group members to freely recall past experiences and ensuring that each participant speaks for at least 10 min.
3rd (week 3)	**Life stage**: Adulthood (25–65 years) **Topic**: spouse, parenting, professional experience etc. **Members**: 1 implementer and 6–7 participants. **Duration**:90 min. **Method**: The implementer will be responsible for guiding and encouraging the group members to freely recall past experiences and ensuring that each participant speaks for at least 10 min.
4th (week 4)	**Life stage**: Old age (over 65 years) **Topic**: philosophy of life Looking back etc. **Members**: 1 implementer and 6–7 participants. **Duration**:90 min **Method**: The implementer will be responsible for guiding and encouraging the group members to freely recall past experiences and ensuring that each participant speaks for at least 10 min.

#### Reminiscence therapy combined with memory specificity training

The process and content of reminiscence therapy is the same as described above, but the researcher will help participants to make their recollections more specific and complete when they recall a particular episode. Prompts that guide recall draw on other research [49] and cover the following areas:

- Details – who/what/where/when.
- Five senses – smell/taste/hear/see/touch.
- Identity – what does this tell us about you as a person?
- Positive themes – what was good about this event for you?
- Generalization of positive themes – does this link to other activities you have done? How?
- Future planning – what steps could you take to repeat this activity?

#### Usual care

All participants will receive usual care, and common nursing home routines are shown in Table 3.

**Table 3:** Routine care

Life Care	Recreational activities
1. Cleanliness and hygiene	1. Tea ceremony
2. Sleep care	2. Handcrafted
3. Dietary care	3. Calligraphy
4. Excretion care	4. Ping pong
5. Safe care	5. Chorus

### Outcome measures

See Table 4 for an overview of all measurement instruments and timepoints for older people.

**Table 4:** Overview of instruments per assessment

Measures	T0	T1	T2	T3	T4	
Demographics	×					
GDS	×	×	×	×	×	
ATM	×	×	×	×	×	
RRS	×	×	×	×	×	
IPA	×	×	×	×	×	
T0: baseline, T1: immediately postintervention, T2: 1month postintervention, T3: 3 months postintervention, T4: 6 months postintervention.						

#### Depressive symptoms in older people

Depressive symptoms in older people will be measured by the Geriatric Depression Scale. This scale was developed by Yesavage [50] et al. as a special depression screening scale for people over 56 years of age, and each examination takes approximately 15 min. It consists of 30 questions and four factors: ① unhappiness ② apathy and anxiety ③ loss of hope and morale ④ memory loss and reduction of social activity. Ten of the 30 items are scored in the reverse direction and 20 in the positive direction. The total score is 0–30, with higher scores indicating more severe depression. Scores of 0–10 are normal, scores of 11–20 indicate mild depression, and scores of 21–30 scores indicate moderate and severe depression. The Cronbach coefficient of the GDS is > 0.85, the retest reliability is > 0.73, and the reliability is > 0.84, with good internal consistency and stability [51].

#### Autobiographical memory

Participants’ autobiographical memory will be measured by the Autobiographical Memory Test. The Autobiographical Memory Test was developed by Williams and Broadbent in 1987 [52] and is a test that is currently widely used in clinical research; this assessment utilizes a cue word technique in which subjects review past personal experiences through cue words that contain five positive cue words, five neutral words, and five negative cue words. Participants will be given two neutral practice words to practice before the formal test. They will be asked to verbally recall memories related to cue words within 1 min during the formal administration of the test, and the implementer will record the recollections in the form of a transcript. The scoring will be performed by two researchers who are not involved in the administration of the test. The two scorers will classify the participants’ recollections into four categories: specific memory, generalized memory, vocabulary association or nonrecollection and nonresponse, and the scoring will be based on the number of cue words recalled by the participants. Since Chinese scholars have translated the cue words in this test into Chinese, composed a vocabulary database and rated the valence of the vocabulary, the Chinese version of the ATM will be used in this study [53], which has a high internal consistency and a reliability test Kappa coefficient of 0.81–0.95 [54]. Cue words that will be used in the assessments and training are shown in Table 5.

**Table 5:** Cue words

pre-test	post-test	follow-up
Strong	Deep Love	Honest
History	Books	Relaxed
Fear	Sadness	Friendly
Security	Interesting	Guilty
Individual	Oval	Nervous
Failure	Reject	Desperate
Excitement	Lively	Patience
Caution	Bread	Agile
Solitude	Doubt	Culture

#### Rumination

Rumination will be measured by the Ruminative Responses Scale (RRS). The scale was developed by Nolen-Hoeksema [55], and the Chinese version was revised by Han Soo [56] et al. The scale consists of 3 dimensions, namely, symptom rumination, compulsive thinking, and reflective rumination, with a total of 22 items. The scale is scored on a 4-point scale, with higher scores indicating more severe ruminative thinking. The scale is widely used in China and has good reliability and validity, with reliability coefficients ranging from 0.76 to 0.89 for each dimension.

#### Social engagement

The purpose of assessing social engagement in this study is to avoid confounding the effects of social engagement and reminiscence therapy on depressive symptoms in older adults. Social engagement will be measured by the Chinese version of the Impact on Participation and Autonomy Questionnaire (IPA). This scale was developed by Cardol [57] et al. and measures the individuals’ own perceived social engagement. In this study, the Chinese version of the IPA, which was revised and adapted for use in Chinese populations by Li Hong et al. [58] et al. was employed, including 25 items in four dimensions: indoor autonomous participation (7 items), family role (7 items), outdoor autonomous participation (5 items), and social life (6 items). Each item was rated on a 5-point Likert scale, with “a lot” being assigned a score of 0 and “very little” being assigned a score of 4. The higher the score, the worse was the level of social engagement.

### Statistical analysis

A database will be established using EpiData, and the data will be entered by two persons and analysed statistically using SPSS 26.0 software. The measurement data such as scale scores will be expressed as the mean ± standard deviation (x ± s). Count data such as general demographic data will be described by the frequency and percentage. The status of each scale score before and after the intervention within each group and the measurement data between multiple groups will be analysed by repeated-measures ANOVA and simple effects analysis if they are normal and have equal variance, and the statistics will be described by median and mean difference (MD). For measures with skewed distribution, median and quartiles will be used to describe the statistics, and Kruskal‒Wallis multiple comparisons will be used to compare the data between and within groups. Intention-to-treat will be used to process the data and P < 0.05 will be considered statistically significant.

### Quality Control

Our study team is an experienced group consisting of a senior expert in the field of geriatric care, three graduate students specializing in geriatric care, and nursing home professional nurses. Experienced nursing home professional nurses and the researcher on the team will be responsible for recruiting participants. Participants who meet the inclusion criteria will be screened, informed of the study details by the researcher, and asked to sign a consent form if they agree to participate. If there are complex clinical issues, our researchers, nursing specialists, and nursing home professional nurses will work together to find solutions.

The investigators will provide uniform and standardized training to all personnel implementing the intervention, and will conduct group training and related assessments on the use of the scale to ensure that implementers are familiar with the content and master the use of the scale, and that it will be tested and collected on the spot to ensure the validity and recall of the questionnaire.

## Discussion

Depression in older people is a global public health problem. According to the World Health Organization, adults between the ages of 55 and 74 years are the people most affected by depression [59]. Late life depression (LLD) is a common disabling disorder, with a prevalence ranging from 9–45% [60], especially during the COVID-19 pandemic. Older people may be more vulnerable to the mental health effects of the pandemic and have a significantly increased risk of dementia, whether directly from the infection itself or indirectly from infection-preventive measures [61]. Cost-effective psychological interventions are urgently needed to improve depression in older adults.

Reduced autobiographical memory with increased overgeneral memory as a cognitive marker of depression is not only evident in individuals with a history of depression, but also predicts subsequent increases in symptoms [62]. Fortunately, studies have shown that this overgeneral memory is not a fixed feature of an individual’s memory style but can be modified [63]. In high-risk populations without a clinical diagnosis, the benefit of memory specificity training may be reflected in preventing further severity of symptoms [64], but its effectiveness alone is modest and short-lived [65].

Reminiscence therapy has received increasing attention as a psychotherapy that has been developed over a long period of time and has a more mature intervention model. It helps older adults gradually recall memories of past experiences and unresolved conflicts through many therapeutic aspects, alleviates negative emotions by integrating all past positive and negative experiences, fosters a sense of security, and helps support the intrinsic capacity of older adults to age healthily [28]. Therefore, we will implement a multicentre, single-blind, three-arm parallel group randomized controlled trial to investigate whether the combination of the two therapies can further confirm the role played by autobiographical memory in the overall improvement of depressive symptoms in older adults while promoting the emergence and implementation of more therapies targeting autobiographical memory.

However, this study still has limitations. First, the sample size is small, and the population is limited to three nursing homes, so the results may not be fully generalizable. Second, the study will be conducted by different team members, and the consistency of implementation may be questionable. Finally, the scales used in the study are partially self-reported scores, which may be subject to some bias.

In conclusion, we have designed a study combining two psychological interventions with the expectation that their combined use would increase the improvement of depressive symptoms in older adults. Based on this evidence, we believe that this intervention program will be able to increase autobiographical memory and improve depressive symptoms in older adults. If the intervention produces significant positive effects, the findings will also potentially provide valuable evidence to facilitate and provide feasible strategies to further promote healthy ageing.

## Data Availability

Not applicable.

## List of abbreviations

ATM: Autobiographical Memory
OGM: Over-general Memory
RT: Reminiscence Therapy
MeST: Memory Specificity Training
